# *Ex vivo* cytokine responses and metabolic reprogramming are associated with γδ T cell differentiation in *Plasmodium falciparum* placental malaria

**DOI:** 10.3389/fimmu.2026.1803384

**Published:** 2026-04-16

**Authors:** Chris Marco Nana Mbianda, Bodin Darcisse Kwanou Tchakounte, Bernard Marie Bitye Zambo, Joséphine Ntankheuh Tchinkou, Derrick Atchombat Nyasse, Balotin Fogang, Rafael José Argüello, Lawrence Ayong, Rosette Megnekou

**Affiliations:** 1 Department of Animal Biology and Physiology, Faculty of Sciences, University of Yaoundé I, Yaoundé, Cameroon; 2 Immunology Laboratory of the Biotechnology Center, University of Yaoundé I, Yaoundé, Cameroon; 3 Department of Microbiology, Faculty of Sciences, University of Yaoundé I, Yaoundé, Cameroon; 4 Malaria Research Unit, Centre Pasteur du Cameroun, Yaoundé, Cameroon; 5 Aix-Marseille University, CNRS, INSERM, CIML, Centre d’Immunologie de Marseille, Marseille, France

**Keywords:** cytokines, metabolic reprogramming, placental malaria, *Plasmodium falciparum*, γδT cells memory

## Abstract

**Background:**

γδ T cells play a key role in modulating immune responses to pregnancy-associated malaria and can enhance vaccine efficacy through their activation and cytotoxic functions. However, the mechanisms guiding γδT cell differentiation in placental malaria (PM) remain poorly understood. We examined *ex vivo* associations between cytokines, metabolic profiles and γδ T-cell differentiation in women with PM.

**Methods:**

A case-control study including 50 women at delivery (21 PM+, 29 PM-) was carried out. Peripheral, placental intervillous space, and cord blood mononuclear cells were isolated, and multiparametric flow cytometry was performed to characterize γδT-cells and memory phenotypes, its subsets (Vδ1^+^, Vδ2^+^, Vδ3^+^), and the expression of exhaustion (TIM-3, PD1) and activation (HLA-DR) markers. *Ex vivo* plasma levels of IL-8, IL-33, and IL-35 were quantified by Luminex assay or ELISA. Immunometabolic profiles were assessed in a subset of 15 samples from uninfected women following cell stimulation with phytohemagglutinin (PHA) by SCENITH assay.

**Results:**

In general, the frequency of γδ T cells and their subsets varied depending on the different blood compartments, with naïve and central memory (CM) phenotypes observed mainly in CBMC, while effector memory (EM) and terminally differentiated effector memory (TEMRA) phenotypes were found mainly in PBMC and IVBMC. *Ex vivo* analyses showed that γδ T cell-modulating cytokine IL-8 were associated, in a compartment-dependent manner, with down-regulation of immunoregulatory markers TIM-3 and PD-1. Interestingly, IL-8 and IL-33 were associated with increased frequency of the TEMRA cell phenotype in peripheral blood, consistent with enhanced differentiation of naïve γδT cells during PM. Metabolic profiling of the different cell types further established an enhanced mitochondrial metabolic activity in predominantly terminally differentiated γδ T cells in PBMC, compared to the mainly glycolytic activities of non-terminally differentiated cells in CBMC and IVBMC.

**Conclusion:**

Placental malaria is associated with a compartment-specific memory γδ T cell differentiation, with cytokines and metabolic reprogramming regulating exhaustion. These findings reveal key regulatory processes that determine the function of γδ T cells during placental malaria, which constitute potential targets for new therapeutic intervention against PM.

## Background

Pregnancy-associated malaria (PAM) remains a major public health concern, with an estimated 12.7 million pregnant women exposed to *Plasmodium falciparum* infection in sub-Saharan Africa (1). Beyond its clinical impact, PAM presents a unique immunological challenge, as the maternal immune system must simultaneously tolerate the semi-allogeneic fetus while developing an effective response against *Plasmodium falciparum* infection. One of the most severe manifestations of PAM is placental malaria (PM), characterized by the sequestration of infected erythrocytes (IEs) within the placental intervillous spaces (2). This sequestration is mediated by the Variant 2 Chondroitin Sulfate A protein (VAR2CSA), a member of the *Plasmodium falciparum* erythrocyte membrane protein 1 (PfEMP1) family, which enables IEs to specifically bind to chondroitin sulfate A (CSA) expressed on the surface of the placental syncytiotrophoblast (3). This interaction allows IEs to escape splenic clearance, leading to their accumulation within the placenta and triggering local inflammation and loss of placental membrane integrity (4). These pathological alterations disrupt maternal-fetal exchanges, promote leukocyte infiltration, and induce the production of cytokines and chemokines, which are associated with poor pregnancy outcomes such as maternal anemia, low birth weight (5, 6), intrauterine growth restriction, and preterm delivery (7, 8). Previous studies, including ours, have shown that plasma concentrations of cytokines and chemokines such as IFN-γ, IL-4, IL-10, IL-8 (CXCL-8), and CXCL-13 are significantly higher in PM+ women (6, 9, 10), reflecting an inflammatory response aimed at controlling. However, excessive cytokine production contributes to pathological inflammation and poor pregnancy outcomes, including maternal anemia and low birth weight (5, 9, 11).

Among the known inflammatory cytokines, IL-8, IL-33, and IL-35 may exhibit complementary immunomodulatory functions during PM. IL-8 (CXCL-8) is a potent chemoattractant that promotes neutrophil recruitment and γδ T cell activation (12). IL-33, a member of the IL-1 cytokine family, acts as an alarmin released upon tissue injury with roles in modulating both innate and adaptive immune responses, including those mediated by γδ T cells (13). Conversely, IL-35 exerts anti-inflammatory effects by promoting regulatory T cell (Treg) differentiation and suppressing pro-inflammatory Th1 and Th17 responses (14). *In vitro* studies have implicated IL-35 in modulating γδ T-cell differentiation, reducing granzyme B expression while enhancing the expression of exhaustion markers such as PD-1 and LAG-3 (15). γδ T cells constitute a distinct subset of cells that bridge innate and adaptive immunity (16, 17). They exhibit antimicrobial, regulatory, and tissue-repair functions, and have recently emerged as important contributors to antimalarial immunity (18, 19). Despite their relatively low frequency in peripheral blood, γδ T cells and its subsets (Vδ1^+^ and Vδ2^+^) differentially redistribute in various blood compartments during PM and have been associated with pregnancy parameters such as gestational age, parity, and gravidity, as well as pregnancy outcomes such as maternal hemoglobin level, and baby birth weight (19). However, the mechanisms associated with the activation and potential differentiation of γδ T cells into various memory cell subtypes remain poorly understood.

Upon antigenic stimulation, activated T cells can differentiate into various memory subsets, including central memory (CM), effector memory (EM), tissue-resident memory (TRM), and terminally differentiated effector memory (TEMRA) cells, each with distinct cytotoxic, proliferative, and homing properties (20, 21). While T cell differentiation is driven by antigenic signals, the surrounding metabolic microenvironment profoundly influences T-cell function. Indeed, T cells engage specific metabolic pathways upon stimulation to support their proliferation and effector activity (22). As such, naïve T cells primarily rely on oxidative phosphorylation (OXPHOS) and fatty acid oxidation (FAO) for ATP generation, whereas effector T cells switch to aerobic glycolysis (“Warburg effect”) to sustain rapid growth and cytokine production (23). New evidence also suggests that cytokines can modulate cellular metabolism, thereby linking immune regulation to bioenergetic reprogramming (24). Although such differentiation processes have been well characterized in cancer and *in vitro* systems, no studies to date have demonstrated *ex vivo* the relationship between plasma levels of IL-8, IL-33, IL-35, metabolic reprogramming and γδ T cells phenotype during placental malaria in women at delivery. Therefore, we hypothesized that these cytokines, in conjunction with nutrient availability, modulate γδ T-cell differentiation in *Plasmodium falciparum* placental malaria in women at delivery, potentially influencing pregnancy outcomes. Understanding these mechanisms may facilitate the development of novel therapeutic interventions against PM, essential for improved maternal health during pregnancy particularly in regions with a high risk of pregnancy-associated *Plasmodium falciparum* malaria.

## Methods

### Study design and sample collection

This case-control (2:3 ratio) study was conducted between March 2022 and May 2023 as previously described (19). The study involved pregnant women residing in urban and suburban areas of Yaoundé, Cameroon, who sought medical care at the Marie Reine Health Center, Etoudi and Marie des Anges Catholic Hospital, Koabang for childbirth. These healthcare centers are located in peripheral districts of Yaoundé where malaria transmission is perennial, with two wet and two dry seasons. A total of 150 blood samples, comprising 50 from each blood compartment (peripheral, placental and cord blood), were collected immediately after delivery from 21 women who tested positive for placental malaria and 29 women who tested negative.

Data on maternal demographics, health status, pregnancy history, gestational details, utilization of intermittent preventive treatment with sulfadoxine-pyrimethamine during pregnancy (IPTp-SP), insecticide-treated bed net usage (ITN), and birth weight were recorded using a standardized questionnaire. Women with pre-existing medical conditions such as preeclampsia, diabetes, toxoplasmosis, hepatitis, syphilis, and positive HIV status were excluded from the study. Birth weight less than 2.5 kg were considered low according to World Health Organization’s guidelines (25). Maternal venous blood as well as placental and cord blood samples were aseptically collected in BD vacutainer tubes just after delivery. Placental tissue fragments were also collected from the maternal side of the placenta and used for placental malaria diagnosis. All blood samples were processed on the day of collection.

### Diagnosis of placental malaria and determination of hemoglobin levels

*Plasmodium falciparum* infection was determined using the Commercialized One Step HRP-II and pLDH RDT rapid diagnostic test (SD Bioline malaria antigen P.f/Pan, Standard Diagnostics Inc, Kyonggi-do, South Korea), and confirmed by thick blood smears microscopy using participant’s venous, placental and cord blood samples. Placental malaria was defined based on the presence of infected erythrocytes in impression smears from placental tissue. Smears were Giemsa-stained and examined by at least two expert microscopists for the presence of malaria parasites. Hemoglobin (Hb) levels in maternal and neonate blood were determined using a hemoglobinometer (ACON Laboratories, INC, San Diego, USA). Women at delivery were considered anemic if Hb level was <11 g/dl, whereas neonates were considered anemic in case of Hb level < 12,5 g/dl (26).

### Isolation of PBMCs, IVBMCs and CBMCs

Cells isolation was performed as previously described (19). Briefly, cells isolated included Peripheral Blood Mononuclear Cells (PBMCs) from maternal venous blood, Intervillous Blood Mononuclear Cells (IVBMCs) from the intervillous space of the placenta, and Cord Blood Mononuclear Cells (CBMCs) from the umbilical cord. Approximately, 15 ml of each blood sample (peripheral, placental or cord blood) was diluted 1:1 in 1X PBS (without Ca^2+^ and Mg^2+^), supplemented with 2% FBS (ThermoFisher Scientific, USA). The diluted samples were layered onto 15 ml Histopaque 1077 (Sigma-Aldrich, USA) in a 50 ml Falcon tube and centrifuged at 800g for 30 minutes at 20 °C without brake. The mononuclear cell layers (PBMC, IVBMC and CBMC) were collected and washed twice with 1X PBS (without Ca^2+^ and Mg^2+^), supplemented with 2% FBS by centrifugation at 600 g for 8 minutes at 20 °C. Cells viability was assessed using the Trypan blue dye exclusion method, and only samples with viability greater than 95% were used in further analyses. Approximately 1×10^6^ isolated PBMCs, IVBMCs or CBMCs were resuspended in 1mL of 1X PBS for surface staining.

### *In vitro* γδ T cells stimulation

A total of 15 uninfected women at delivery were sampled and mononuclear cells from each blood compartment (PBMC, IVBMC, CBMC) were isolated. These cells were cultured for 7 days in an incubator at 37 °C and 5% CO2 using complete RPMI 1640 culture medium (Gibco, cat# 11020021, USA), supplemented with 100 IU/mL rhIL-2 (Thermo Fisher Scientific, cat# PHC0021, USA), 10% FBS (Gibco, cat# 10082147, USA), 1% Penicillin-Streptomycin (Gibco Thermo Fisher Scientific, cat# 15140122, USA), 55µL β-Mercaptoethanol (Sigma Aldrich, cat# M6250-10ML, France) and 5 µg/mL phytohemagglutinin (PHA) (Gibco Thermo Fisher Scientific, cat# 10576015, USA). Following this period, the cells were washed and analyzed by SCENITH-based flow-cytometry.

### SCENITH™ metabolic profiling

A total of 15 samples from uninfected women at delivery were stimulated to investigate the metabolic capacities and dependencies of γδ T cells. The SCENITH™ kit, containing all necessary reagents (including anti-puromycin, clone R4743L-E8) and detailed protocols (23), was used to analyze the metabolic profile of γδ T cells. Briefly, 9 × 10^5^ cells from each blood compartment (PBMCs, IVBMCs, and CBMCs) were plated in duplicates in complete RPMI 1640 medium in a 24-well plate and treated with Puromycin (10 µg/mL), DMSO (Co), 2-deoxy-glucose (2DG; 100 mM), oligomycin (O; 1 µM), a combination of 2DG and oligomycin (DGO) or harringtonine (H; 2 µg/mL) for 40 min at 37 °C, 5% CO_2_. Following the incubation, cells were washed in cold FACS buffer and further incubated for 15 min with a fixable viability dye. Cells were again washed, incubated with Fc Block (BioLegend, cat#422302, USA), and stained with conjugated antibodies for 30 min at 4 °C in FACS Buffer. Cells were then fixed and permeabilized with the Foxp3 staining kit according to the manufacturer’s instructions (ThermoFisher Scientific). Intracellular staining with Alexa Fluor 488-conjugated anti-puromycin monoclonal antibody (clone R4743L-E8) was performed by incubating cells during 1 hour at 4 °C in Permeabilization Buffer (ThermoFisher Scientific) and analyzed by flow cytometry.

### Surface staining

Surface staining of PBMCs, IVBMCs, CBMCs and PHA-stimulated cells were performed in 96-well V bottom shaped Microplate. Cells were washed with 200 µl of FACS buffer (1X PBS without Ca^2+^ and Mg^2+^, 5% FBS, 5mM EDTA), re-suspended in 50 µl of human FcR Blocking Reagent (Miltenyi Biotech, USA), and incubated for 10 min at 4 °C in the dark. After washing, the pellet was re-suspended in 50 µl of antibody staining mix and incubated for 30 min at 4 °C in the dark. Cells were then washed with 200 µl of FACS buffer at 1800 rpm for 3 min and re-suspended in 200 µl of FACS buffer prior to acquisition. The following monoclonal antibodies were used for surface staining: Brilliant Violet (BV) 510 labelled anti-CD3 (clone UCHT1), Allophycocyanin (APC) labelled anti-CD27 (clone M-T271), Fluorescein Isothiocyanate (FITC) labelled anti-CD45RA (clone HI100) (BD Biosciences, USA); Phycoerythrin (PE) labelled anti-TCRγδ PE (clone B1); APC-Cyanine 7 (APC-Cy7) labelled anti-TCR V delta 2 (clone B6), BV421 labelled anti-TIM-3 (clone F38-2E2); PE-Cy7 labelled anti-TCR V delta 1 (clone TS8.2), Fluorescein Isothiocyanate (FITC) labelled anti-HLA-DR (clone L243)(ThermoFisher Scientific, USA). Data were acquired within 2 hours of staining on a BD FACS Canto II flow cytometer equipped with a BD FACSDiva software version 6.1.3 (Becton Dickinson, USA).

### Flow cytometry data analysis

**Figure 1:**
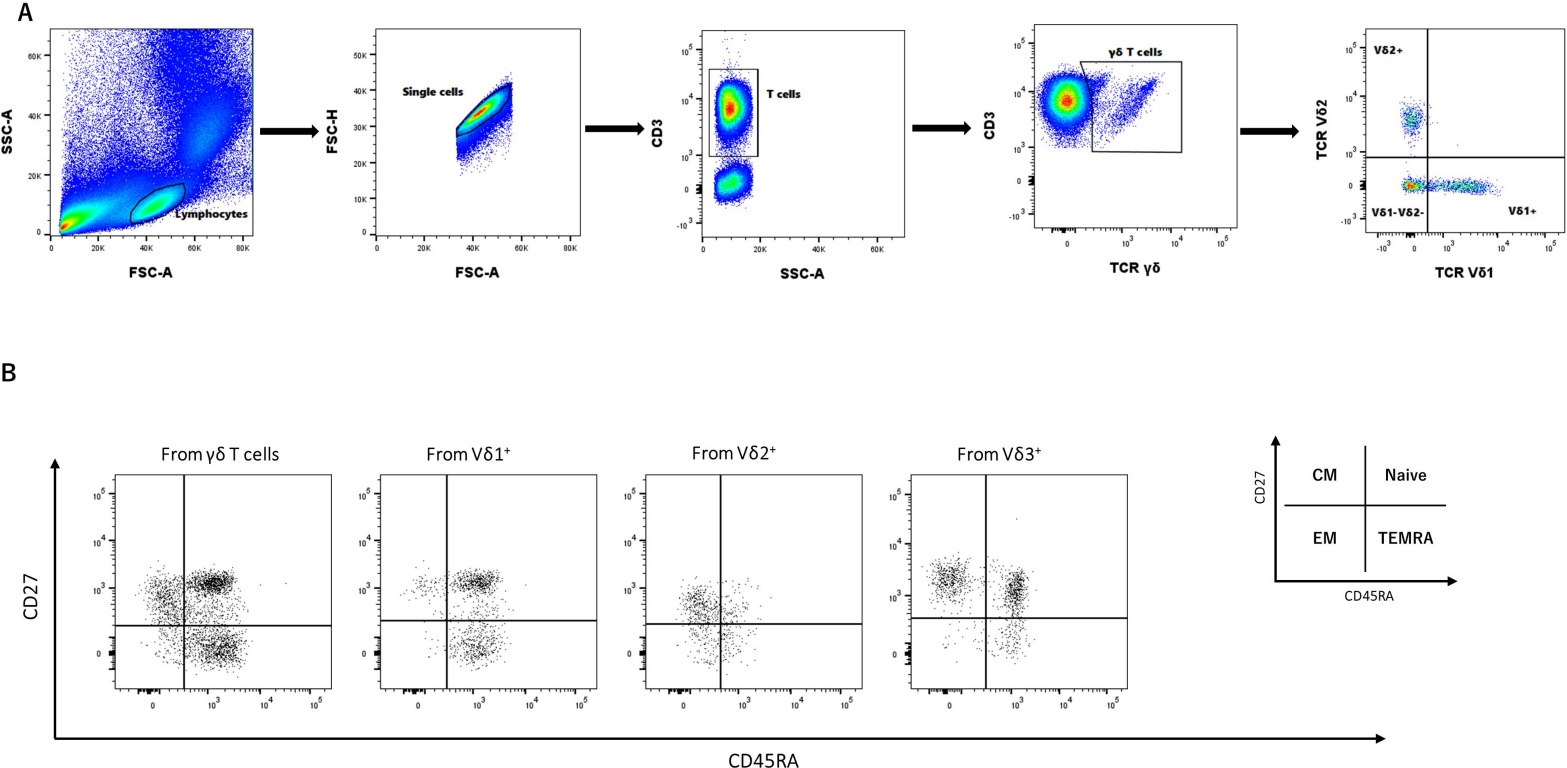
Gating strategy of different memory γδ T cells and their subsets. **(A)** Samples were initially gated on lymphocytes based on SSC-A/FSC-A gating followed by Single cell gating using FSC-H/FSC-A. T cells were gated from singlets as CD3^+^ cells, followed by γδ T cell gating as TCRγδ^+^. Different subsets of γδ T cells were characterized using TCR Vδ1 and TCR Vδ2 markers as either Vδ1^+^, Vδ2^+^ or Vδ1^-^Vδ2^-^ (Vδ3^+^). Vδ3 cells were defined as CD3^+^TCRγδ^+^Vδ1^−^Vδ2^−^ cells. As a Vδ3-specific antibody was not available, this gating identifies a Vδ3-enriched population but may also include other minor γδ T-cell subsets. Therefore, it should be interpreted as a surrogate rather than a definitive identification. **(B)** Memory cells were gated within different populations and subsets using CD45RA and CD27 markers. naïve cells: CD45RA^+^CD27^+^; Central Memory (CM) cells: CD45RA^-^CD27^+^; Effector Memory (EM) cells: CD45RA^-^CD27^-^; Terminally Differentiated Effector Memory (TEMRA) cells: CD45RA^+^CD27^-^.

Flow cytometry data were analyzed using FlowJo software version 10.10.0 (Tree Star, Inc) and OMIQ. Color compensation was performed with beads stained for each fluorochrome. Only samples with at least 100–000 CD3^+^ single cells were included in the analysis. The FlowAI algorithm was applied to automatically clean the FCS data of adverse events. A lymphocyte gate was set based on SSC-A/FSC-A, followed by single cell gating based on FSC-H/FSC-A. T cells were gated from singlets as CD3+ cells and γδ T cells were gated from CD3+ as TCRγδ+. Different subsets of γδ T cells were characterized using TCR Vδ1 and TCR Vδ2 markers as Vδ1^+^, Vδ2^+^ and Vδ1^-^Vδ2^-^ (Vδ3^+^). Vδ3 cells were defined as CD3^+^TCRγδ^+^Vδ1^−^Vδ2^−^ cells, which may include other minor γδ T-cell subsets and therefore serving as surrogate population for the targeted Vδ3 cells. Naive and memory cells were gated within different populations and subsets using CD45RA and CD27 markers as naive cells: CD45RA^+^CD27^+^, Central Memory (CM):CD45RA^-^CD27^+^, Effector Memory (EM): CD45RA^-^CD27^-^, Terminally Differentiated Effector Memory (TEMRA):CD45RA^+^CD27^-^ (**Figure 1**).

### *Ex vivo* cytokine quantification

Enzyme-linked immunosorbent assay (ELISA) kit (Human IL-35 DuoSet ELISA kit, R&D System, cat# DY6456-05, USA) was used to assess IL-35 plasma levels according to the manufacturer’s instructions. Meanwhile, IL-8 and IL33 plasma levels were assessed using Multianalyte platform assay kit (Human Premixed multi-analyte kit, cat# LXSAHM-05, R&D SYSTEMS, Inc. Minneapolis, MN, USA) according to the manufacturer’s instructions.

### Statistical analysis

Statistical analyses were performed using GraphPad Prism version 8.4.3. Data normality was assessed for each variable using the Shapiro-Wilk test. A p-value > 0.05 indicated normal distribution. Normally distributed data were reported as means with standard deviations, while non-normally distributed data were reported as medians with interquartile ranges. Comparison between two unpaired groups were performed using Student’s t-test for normally distributed data or Mann-Whitney rank sum for non-normally distributed data. Spearman rank order correlation test was used to assess the correlation between two quantitative variables. To control for false discoveries arising from multiple testing, the Benjamini-Hochberg false discovery rate (FDR) correction was applied across all performed correlation analyses. Adjusted q-values less than 0.05 were considered statistically significant. Proportions were compared using Fisher’s exact test. *P values* < 0.05 were considered statistically significant.

## Results

### General characteristics of the study population

The study included 29 placental malaria-negative (PM-) and 21 placental malaria-positive (PM+) women. **Table 1** summarizes the basic demographic, clinical, and obstetrical characteristics of the study population. No significant difference was observed between PM+ and PM- women in terms of participant’s age, parity, gravidity and gestational age (p>0.05). The median maternal hemoglobin level was significantly lower (p=0.002) in PM+ compared to PM- women. Similarly, the prevalence of maternal anemia was significantly higher in PM+ than in PM- women (p=0.003). In contrast, fetal hemoglobin levels (15.66 g/dL versus 14.50 g/dL), as well as the prevalence of fetal anemia (50% versus 38.10%) did not differ significantly between PM+ and PM- women (p=0.221 and p=0.563, respectively). Babies born from placental infected women (PM+) had significantly lower birth weight than those from non-infected placenta (PM-) group (p=0.014). Regarding the proportion of women who had taken IPTp-SP (88.89% versus 85.71%) or used ITNs (82.14% versus 90.48%) during pregnancy, no significant difference (p>0.999 and p=0.683, respectively) was observed between the PM- and PM+ groups.

**Table 1:** General characteristics of the study population.

Variables	All women N=50	PM- women n=29	PM+ women n=21	p values
Age in years (mean ± SD)	26.44 ± 5.155	27.03 ± 5.402	25.65 ± 4.801	0.343
Parity [median and 25% - 75% IQR]	2 [1 - 4]	2 [1 - 4]	2 [1 - 2.5]	0.086
Gravidity [median and 25% - 75% IQR]	3 [1.75 - 4]	3 [2 - 4]	2 [1 - 4]	0.535
Maternal hemoglobin levels in g/dL median [25% - 75% IQR]	12 [10.50 – 13,17]	12.67 [11.66 – 14.25]	10.67 [10.33 - 12.33]	0.002
Percentage of maternal anemia (%)	14/49 (28.57)	3/28 (10.71)	11/21 (52.38)	0.003
Gestational age median [25% - 75% IQR]	39 [38 – 40.5]	39 [38 - 41]	39 [37.25 - 40]	0.212
Fetal hemoglobin levels in g/dL (mean ± SD)	14.74 ± 1.978	14.50 ± 2.01	15.66 ± 1.90	0.221
Percentage of fetal anemia (%)	29/49 (44.90)	14/28 (50)	8/21 (38,10)	0.563
Baby birth weight (mean g ± SD)	3229 ± 427.8	3353 ± 397.9	3045 ± 413.1	0.014
IPTp-SP usage (%)	42/48 (87.5)	24/27 (88.89)	18/21 (85.71)	>0.999
ITNs usage (%)	42/49 (85.71)	23/28 (82.14)	19/21 (90.48)	0.683

PM-, Placental Malaria negative women; PM+, Placental Malaria positive women; IPTp-SP, Intermittent preventive treatment during pregnancy with sulphadoxine-pyrimethamine; ITNs, Insecticide-treated bed net; IQR, Interquartile ranges; SD, Standard deviation. Level of significance, p<0.05.

### Distribution of memory γδ T cell phenotypes according to blood compartments

**Figure 2:**
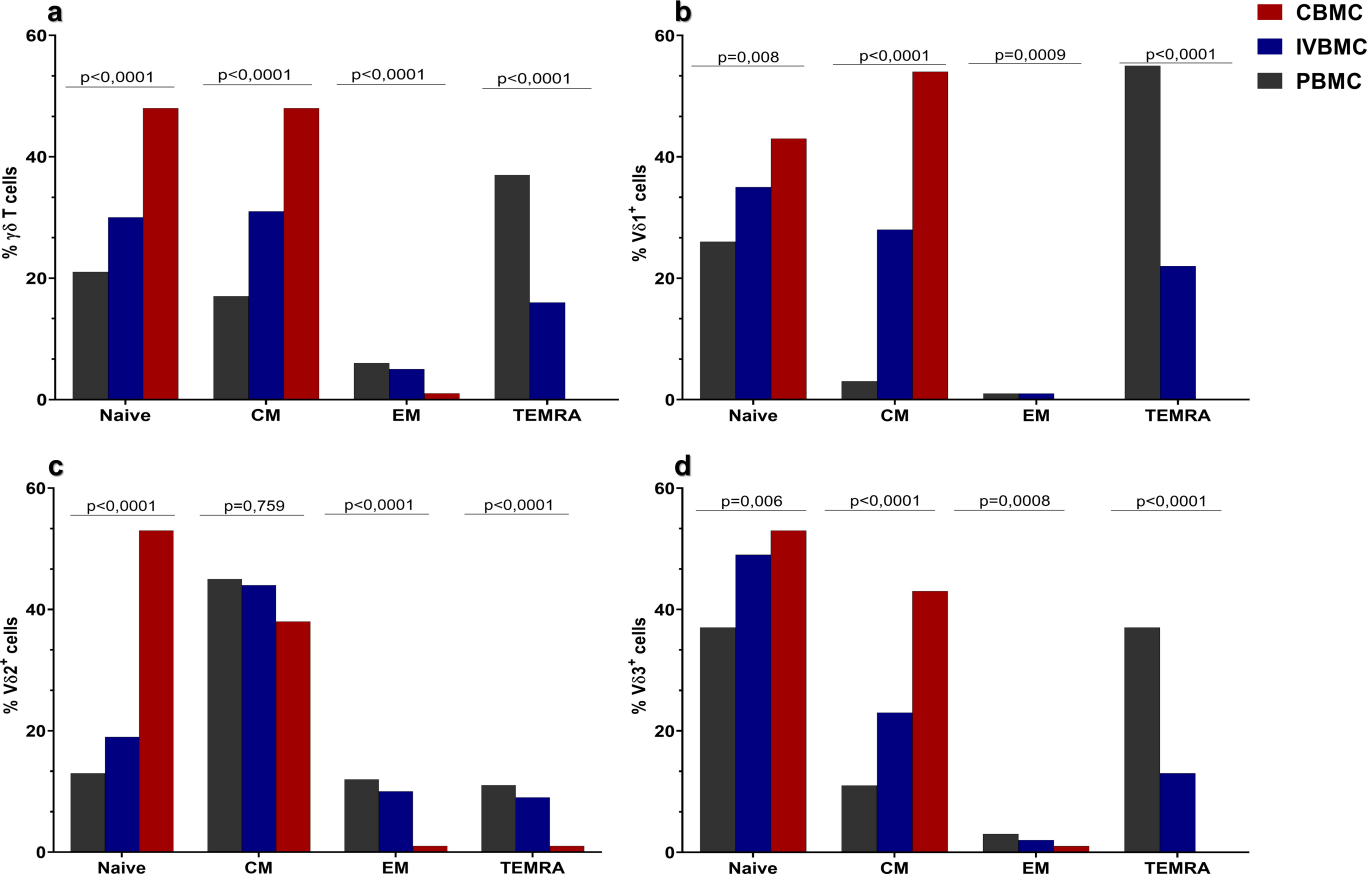
Frequencies of different memory γδ T cells, Vδ1^+^, Vδ2^+^ and Vδ1^-^Vδ2^-^ subsets in PBMC, IVBMC and CBMC. Frequencies of naïve cells and other memory subsets in **(a)** γδ T cells, **(b)** Vδ1^+^ cells, **(c)** Vδ2^+^ cells, **(d)** Vδ3^+^ cells were compared between PBMC (in black), IVBMC (in blue) and CBMC (in red) using Mann-Whitney test. PBMC, Peripheral blood mononuclear cell; IVBMC, Intervillous blood mononuclear cell; CBMC, Cord blood mononuclear cell. Naïve, CD45RA^+^CD27^+^; CM (Central Memory) cells: CD45RA^-^CD27^+^; EM (Effector Memory) cells: CD45RA^-^CD27^-^; TEMRA (Terminally Differentiated Effector Memory) cells: CD45RA^+^CD27^-^.

Building on our previous study, which showed that the frequency of γδ T cells—including their subsets—and their activation (HLA-DR) and exhaustion (TIM-3, PD-1) phenotypes vary across blood compartments (19), we next investigated whether the distribution of memory γδ T cells and their subsets (Vδ1^+^, Vδ2^+^, Vδ3^+^) is similarly compartment-dependent. Memory γδ T cell subtypes were quantified by multiparametric flow cytometry in each participant’s peripheral, placental intervillous space, and cord blood samples. As shown in **Figure 2**, naïve γδ T cells and its subsets (naïve Vδ1^+^, Vδ2^+^ and Vδ3^+^) were predominantly found in CBMC, followed by IVBMC and PBMC. The frequency of Central Memory (CM) γδ T cells, Vδ1^+^, and Vδ3^+^ were similarly most abundant in CBMC follow by IVBMC and PBMC (**Figure 2**). No significant difference in CM Vδ2^+^ cell population was observed between the three blood compartments. Interestingly, the frequencies of Effector Memory (EM) and Terminally Differentiated Effector Memory cells (TEMRA) γδ T cells and subsets were mainly present in PBMC, followed by IVBMC and CBMC (**Figure 2**). These result support the hypothesis that, at birth, infants possess a predominantly incompletely differentiated repertoire of immune cells compared to adults.

### *Plasmodium falciparum* Placental malaria differentially modulates memory γδ T cell phenotype frequencies

**Figure 3:**
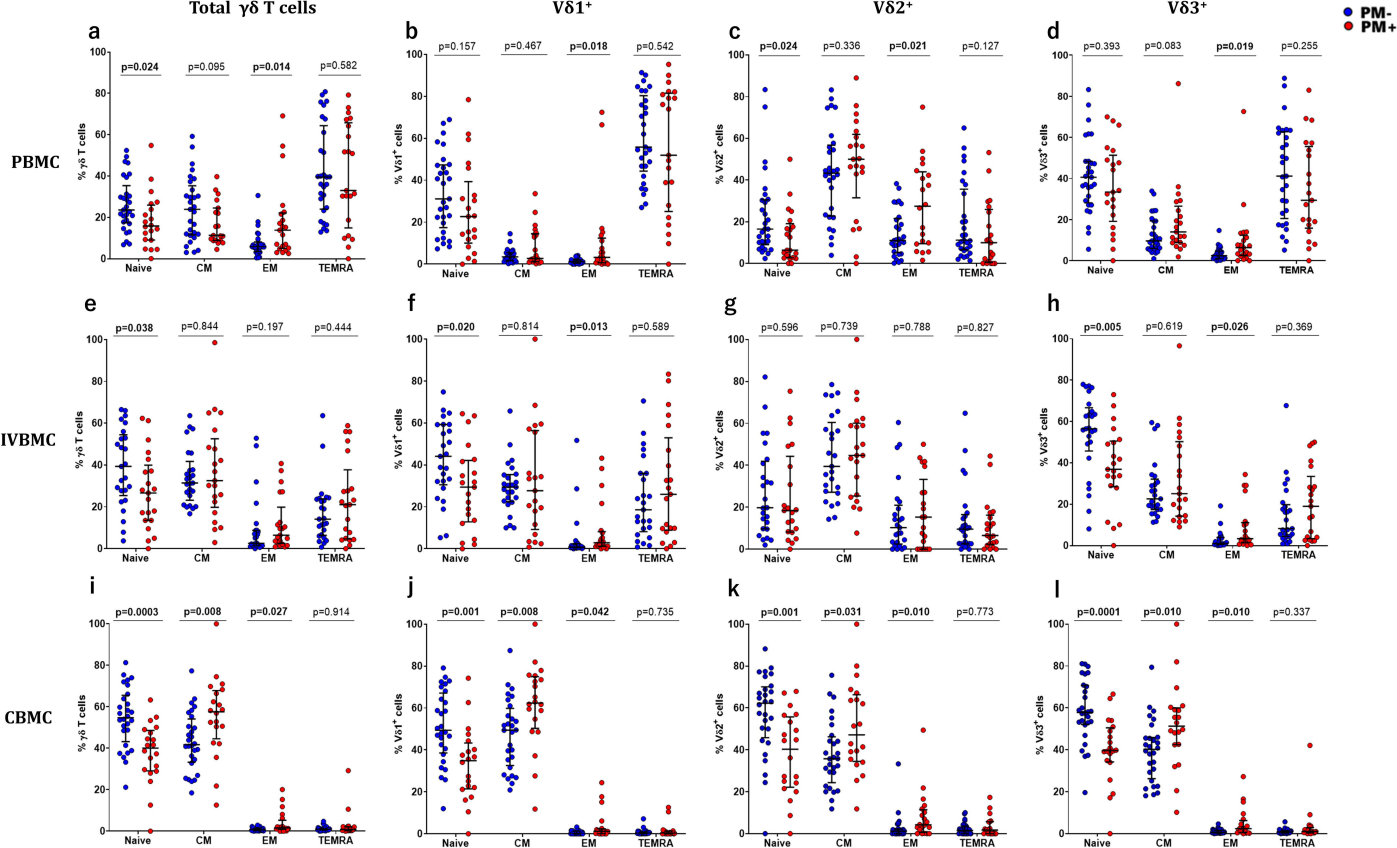
Frequencies of different subsets of memory γδ T cells in PBMC, IVBMC, CBMC compared between PM- and PM+ women. Memory γδ T cells were analyzed by multiparametric flow cytometry in PBMC of PM- (in blue) and PM+ (in red) women, and compared using Mann-Whitney test. Blood compartments (PBMC, IVBMC, and CBMCs) showing memory cell frequencies in γδ T cells **(a, e, i)**, Vδ1^+^ **(b, f, j)**, Vδ2^+^ (**c, g, k)** and, Vδ3^+^ **(d, h, l)** subsets. PM-, Placental Malaria negative women; PM+, Placental Malaria positive women; PBMC, Peripheral blood mononuclear cell; IVBMC, Intervillous blood mononuclear cell; CBMC, Cord blood mononuclear cell; Naïve, CD45RA^+^CD27^+^; CM (Central Memory) cells, CD45RA^-^CD27^+^; EM (Effector Memory) cells, CD45RA^-^CD27^-^; TEMRA (Terminally Differentiated Effector Memory) cells, CD45RA^+^CD27^-^. Each dot represents a single individual.

To investigate whether *Plasmodium falciparum* placental infection influences γδ T cell memory profiles, we compared the frequencies of memory γδ T cell subsets in PBMC, IVBMC, and CBMC. We observed that in PBMC, the frequencies of naïve γδ T cells and Vδ2^+^ cell were lower, while EM γδ T cells, and its subsets (Vδ1^+^, Vδ2^+^, and Vδ3^+^), were higher in PM+ women compared to PM- women (**Figures 3a-d**). Moreover, Placental parasitemia correlated negatively with the frequencies of naïve γδ T cells and Vδ2^+^ cell, but positively with EM γδ T cells and subsets (**Table 2**). TEMRA Vδ2^+^ cells correlated negatively with peripheral parasitemia, while CM cells showed no significant correlations. While these correlations reached nominal significance (p < 0.05), none remained significant after controlling for multiple testing using the Benjamini-Hochberg FDR correction (**Table 2**).

**Table 2:** Correlations between different subsets of memory γδ T cells in PBMC and different parasitemias.

PBMC		% IRBCs of placenta tissue impression smear			Placental blood parasitemia			Peripheral blood parasitemia		
		r_s_	p value	q value	r_s_	p value	q value	r_s_	p value	q value
TCR γδ^+^	Naïve	-0.375	0.008	0.077	-0.351	0.013	0.216	-0.312	0.033	0.127
	CM	-0.218	0.137	0.211	-0.175	0.234	0.536	-0.161	0.285	0.449
	EM	0.351	0.013	0.077	0.275	0.053	0.318	0.369	0.010	0.083
	TEMRA	-0.071	0.623	0.638	-0.053	0.715	0.800	-0.114	0.439	0.471
Vδ1^+^	Naïve	-0.267	0.061	0.139	-0.268	0.060	0.318	-0.208	0.155	0.276
	CM	0.109	0.450	0.514	0.046	0.750	0.800	0.114	0.441	0.471
	EM	0.330	0.019	0.077	0.133	0.358	0.572	0.279	0.055	0.146
	TEMRA	-0.068	0.638	0.638	-0.034	0.812	0.812	-0.127	0.389	0.471
Vδ2^+^	Naïve	-0.313	0.027	0.085	-0.152	0.292	0.572	-0.233	0.111	0.223
	CM	0.198	0.168	0.224	0.056	0.698	0.800	0.150	0.309	0.449
	EM	0.305	0.033	0.089	0.252	0.081	0.323	0.370	0.010	0.083
	TEMRA	-0.237	0.098	0.196	-0.138	0.338	0.572	-0.298	0.040	0.127
Vδ3^+^	Naïve	-0.209	0.145	0.211	-0.176	0.22	0.536	-0.260	0.074	0.169
	CM	0.218	0.128	0.211	0.095	0.513	0.684	0.118	0.423	0.471
	EM	0.336	0.017	0.077	0.204	0.155	0.496	0.338	0.019	0.101
	TEMRA	-0.117	0.417	0.513	-0.111	0.444	0.646	-0.087	0.558	0.558

PBMC, Peripheral blood mononuclear cells; %IRBC, percentage of infected red blood cells; Naïve cells, CD45RA^+^CD27^+^; CM (Central Memory), CD45RA^-^CD27^+^; EM (Effector Memory), CD45RA^-^CD27^-^; TEMRA (Terminally Differentiated Effector Memory), CD45RA^+^CD27^-^; r_s_, Spearman’s Rank correlation coefficient. p-values were obtained from Spearman correlation tests. q-values represent p-values adjusted for multiple comparisons using the Benjamini-Hochberg false discovery rate (FDR) method. Statistical significance was defined as q < 0.05.

In IVBMC, the frequencies of naïve γδ T cells, Vδ1^+^, and Vδ3^+^ cells were lower in PM+ women compared to PM- women, while only the EM Vδ1^+^ and Vδ3^+^ cells were higher in PM+ (**Figures 3e, f, h**). Parasitemia from placental tissue smears correlated negatively with naïve γδ T cell, Vδ1^+^ and Vδ3^+^ cell frequencies, and positively with EM Vδ1^+^ and Vδ3^+^ cells (**Table 3**). Peripheral and placental blood parasitemia showed similar trends, with significant negative correlations with the frequency of naïve Vδ3^+^ and positive correlations with the frequency of EM Vδ3^+^ cells (**Table 3**). Although several correlations reached nominal significance (p < 0.05), none remained statistically significant after correction for multiple testing using the Benjamini-Hochberg false discovery rate (FDR) correction (q ≥ 0.05) (**Table 3**).

**Table 3:** Correlations between different subsets of memory γδ T cells in IVBMC and different parasitemias.

IVBMC		% IRBCs of placenta tissue impression smear			Placental blood parasitemia			Peripheral blood parasitemia		
		r_s_	p value	q value	r_s_	p value	q value	r_s_	p value	q value
TCRγδ^+^	Naïve	-0.346	0.019	0.099	0.978	0.056	0.272	-0.172	0.263	0.443
	CM	-0.028	0.852	0.875	0.297	0.306	0.445	-0.186	0.227	0.443
	EM	0.187	0.212	0.566	0.236	0.117	0.311	0.168	0.277	0.443
	TEMRA	0.141	0.349	0.621	0.013	0.271	0.439	0.184	0.232	0.443
Vδ1^+^	Naïve	-0.365	0.013	0.099	-0.313	0.034	0.272	-0.233	0.127	0.443
	CM	-0.112	0.459	0.668	-0.257	0.085	0.272	-0.21	0.171	0.443
	EM	0.298	0.044	0.142	0.140	0.355	0.473	0.26	0.088	0.443
	TEMRA	0.122	0.419	0.668	0.200	0.183	0.417	0.142	0.359	0.522
Vδ2^+^	Naïve	-0.159	0.293	0.585	0.978	0.209	0.418	-0.068	0.663	0.757
	CM	0.041	0.786	0.875	0.297	0.625	0.666	-0.088	0.57	0.722
	EM	0.039	0.797	0.875	0.236	0.42	0.517	-0.008	0.959	0.959
	TEMRA	-0.028	0.853	0.875	0.013	0.925	0.925	-0.055	0.724	0.772
Vδ3^+^	Naïve	-0.413	0.004	0.070	-0.264	0.076	0.272	-0.315	0.037	0.300
	CM	0.024	0.875	0.875	-0.098	0.519	0.593	-0.084	0.587	0.722
	EM	0.332	0.026	0.103	0.269	0.074	0.272	0.345	0.023	0.300
	TEMRA	0.170	0.260	0.585	0.165	0.275	0.439	0.196	0.202	0.443

IVBMC, Intervillous blood space mononuclear cells; %IRBC, percentage of infected red blood cells; Naïve cells, CD45RA^+^CD27^+^; CM (Central Memory), CD45RA^-^CD27^+^; EM (Effector Memory), CD45RA^-^CD27^-^; TEMRA (Terminally Differentiated Effector Memory), CD45RA^+^CD27^-^; r_s_, coefficient of correlation. p-values were obtained from Spearman correlation tests. q-values represent p-values adjusted for multiple comparisons using the Benjamini-Hochberg false discovery rate (FDR) method. Statistical significance was defined as q < 0.05.

Interestingly, in CBMC, PM+ women had significantly lower naïve γδ T cells including Vδ1^+^, Vδ2^+^, Vδ3^+^ subsets, while CM and EM fractions of these cells were higher compared ton PM- women (**Figures 3i–l**). Moreover, parasitemia (placenta tissue impression, placenta and peripheral blood) negatively correlated with naïve γδ T cells and subsets, and positively with CM and EM subsets (**Table 4**). No significant differences were observed in terms of TEMRA cell frequencies between the PM+ and PM- women groups. Importantly, these correlations remained statistically significant after correction for multiple testing using the Benjamini-Hochberg false discovery rate (FDR) correction (**Table 4**). Overall, these results suggest that *Plasmodium falciparum* placental infection is associated with a shift in naïve γδ T cell populations into Central and/or Effector Memory phenotypes, in a manner influenced by parasitemia, across all examined blood compartments.

**Table 4:** Correlations between different subsets of memory γδ T cells in CBMC and parasitemias.

CBMC		% IRBCs of placenta tissue impression smear			Placental blood parasitemia			Peripheral blood parasitemia		
		r_s_	p value	q value	r_s_	p value	q value	r_s_	p value	q value
**TCRγδ^+^**	**Naïve**	**-0.523**	**0.00014**	**0.001**	**-0.423**	**0.003**	**0.007**	**-0.476**	**0.001**	**0.002**
	**CM**	**0.413**	**0.004**	**0.009**	0.297	0.040	0.062	**0.341**	**0.018**	**0.026**
	**EM**	**0.324**	**0.025**	**0.033**	**0.320**	**0.027**	**0.048**	**0.342**	**0.017**	**0.026**
	**TEMRA**	-0.010	0.947	0.947	0.074	0.618	0.706	0.148	0.317	0.362
**Vδ1^+^**	**Naïve**	**-0.519**	**0.00015**	**0.001**	**-0.488**	**0.0004**	**0.003**	**-0.531**	**0.00011**	**0.001**
	**CM**	**0.419**	**0.003**	**0.009**	**0.335**	**0.020**	**0.040**	**0.385**	**0.007**	**0.014**
	**EM**	**0.368**	**0.010**	**0.016**	**0.513**	**0.0002**	**0.003**	**0.481**	**0.001**	**0.002**
	**TEMRA**	-0.032	0.831	0.930	0.061	0.682	0.728	0.103	0.487	0.520
**Vδ2^+^**	**Naïve**	**-0.509**	**0.0002**	**0.001**	**-0.478**	**0.001**	**0.003**	**-0.529**	**0.00011**	**0.001**
	**CM**	**0.329**	**0.022**	**0.032**	0.294	0.043	0.062	**0.311**	**0.031**	**0.042**
	**EM**	**0.385**	**0.007**	**0.012**	**0.348**	**0.015**	**0.035**	**0.404**	**0.004**	**0.010**
	**TEMRA**	0.024	0.871	0.930	-0.028	0.852	0.852	0.094	0.526	0.526
**Vδ3^+^**	**Naïve**	**-0.535**	**<0.0001**	**0.001**	**-0.427**	**0.002**	**0.007**	**-0.486**	**0.0005**	**0.002**
	**CM**	**0.393**	**0.006**	**0.011**	0.279	0.055	0.074	**0.341**	**0.018**	**0.026**
	**EM**	**0.400**	**0.005**	**0.011**	**0.428**	**0.002**	**0.007**	**0.503**	**0.0003**	**0.001**
	**TEMRA**	0.161	0.274	0.338	0.195	0.184	0.226	0.278	0.056	0.068

CBMC, Cord blood mononuclear cells; %IRBC, percentage of infected red blood cells; Naïve cells, CD45RA^+^CD27^+^; CM (Central Memory), CD45RA^-^CD27^+^; EM (Effector Memory), CD45RA^-^CD27^-^; TEMRA (Terminally Differentiated Effector Memory), CD45RA^+^CD27^-^; r_s_, coefficient of correlation. p-values were obtained from Spearman correlation tests. q-values represent p-values adjusted for multiple comparisons using the Benjamini-Hochberg false discovery rate (FDR) method. Statistical significance was defined as q < 0.05. Bold values indicate significance.

### Plasma levels IL-8, IL-33 and IL-35 cytokines associate with γδ T cell differentiation

**Figure 4:**
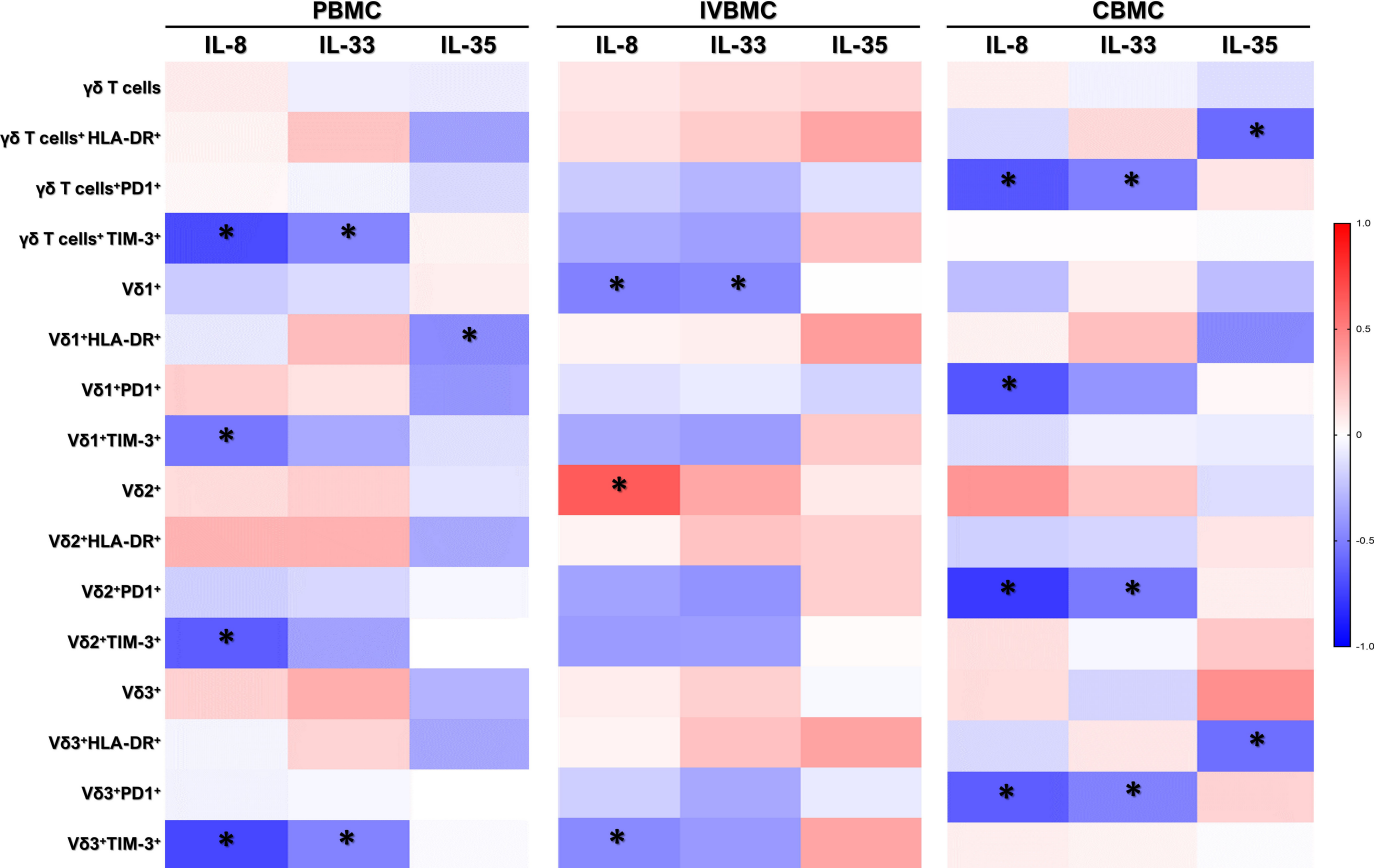
Heatmap depicting the correlation between *ex vivo* IL-8, IL-33, IL-35 cytokine levels and γδ T cell profiles in PBMC, IVBMC, CBMC. Correlation between cytokine levels (IL-8, IL-33, IL-35) and γδ T cell frequency in different blood compartments were determined by Sperman’s rank Order correlation analysis. PBMC, Peripheral blood mononuclear cell; IVBMC, Intervillous blood mononuclear cell; CBMC, Cord blood mononuclear cell; Naïve, CD45RA^+^CD27^+^; CM (Central Memory) cells, CD45RA^-^CD27^+^; EM (Effector Memory) cells, CD45RA^-^CD27^-^; TEMRA (Terminally Differentiated Effector Memory) cells: CD45RA^+^CD27^-^. The asterisks indicate significance. Color intensity indicates correlations: red indicates positive correlations, white indicates no correlation, and blue indicates negative correlations. To control for false discoveries arising from multiple testing, the Benjamini-Hochberg false discovery rate (FDR) correction was applied across all performed correlation analyses. Adjusted q-values < 0.05 were considered statistically significant.

**Figure 5:**
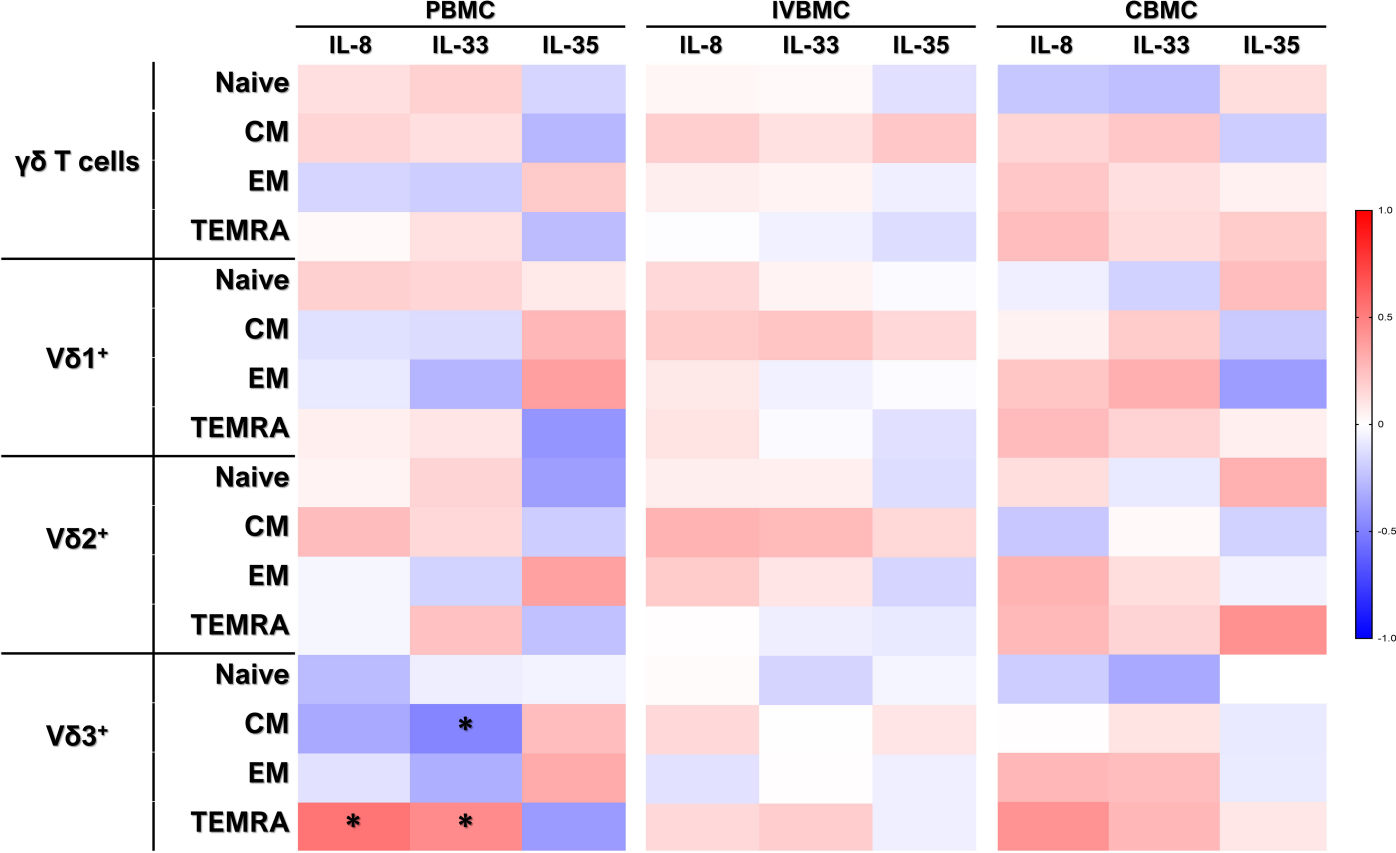
Heatmap representing the correlation between ex vivo cytokines IL-8, IL-33, IL-35 and memory γδ T cell profiles in PBMC, IVBMC, CBMC. Correlation between cytokine levels (IL-8, IL-33, IL-35) and memory γδ T cell frequency in different blood compartments were determined by Sperman’s rank Order correlation analysis. PBMC, Peripheral blood mononuclear cell; IVBMC, Intervillous blood mononuclear cell; CBMC: Cord blood mononuclear cell; Naïve, CD45RA^+^CD27^+^; CM (Central Memory) cells, CD45RA^-^CD27^+^; EM (Effector Memory) cells, CD45RA^-^CD27^-^; TEMRA (Terminally Differentiated Effector Memory) cells: CD45RA^+^CD27^-^. The asterisks indicate significance. Color intensity indicates correlations: red indicates positive correlations, white indicates no correlation, and blue indicates negative correlations. To control for false discoveries arising from multiple testing, the Benjamini-Hochberg false discovery rate (FDR) correction was applied across all performed correlation analyses. Adjusted q-values < 0.05 were considered statistically significant.

During Plasmodium falciparum placental infection, plasma cytokines and chemokines are induced, contributing to both protective immune responses and pregnancy outcomes, and play a critical role in Th1/Th2 responses and differentiation (5, 6). To determine whether cytokines associated with malaria infection induce γδ T cell differentiation, we analyzed *ex vivo* the relationship between plasma levels of some cytokines (IL-8, IL-33, IL-35) and γδ T cell, its subsets and their memory cell phenotypes in PM+ women (**Figures 4**, **5**). In peripheral blood, plasma levels of the pro-inflammatory cytokines IL-8 and IL-33 correlated negatively with TIM-3 expression in total γδ T cells and Vδ3^+^ subset. Only IL-8 correlated negatively with TIM-3 expression in Vδ1^+^ and Vδ2^+^ subsets. Moreover, IL-35 levels negatively correlated with HLA-DR expression in Vδ1^+^ subset. After adjusting for multiple comparisons using the Benjamini-Hochberg FDR method, only IL-8 showed significant associations (q < 0.05). IL-33 and IL-35 did not show any significant associations after FDR correction, even though some nominal p-values were < 0.05, indicating these are likely false positives. These results suggest that, in peripheral blood, IL-8 may contribute to γδ T cell differentiation.

In placental intervillous blood, IL-8 and IL-33 plasma levels negatively correlated with the frequencies of Vδ1^+^ subset, and positively (IL-8 in particular, and to a lesser extent, IL-33) with the frequencies of Vδ2^+^. Moreover, IL-8 plasma levels correlated negatively with TIM-3 expression in Vδ3^+^ cells (**Figure 4**). Importantly, only the correlation between IL-8 plasma levels and Vδ2+ frequency remained significant after FDR correction. Overall, IL-8 appears to be the primary cytokine influencing γδ T cell subset distribution in the placenta.

In cord blood, IL-8 plasma levels significantly correlated with PD-1 expression on γδ T cells, including Vδ1+, Vδ2+, and Vδ3+ subsets (**Figure 4**), and these correlations remained significant after FDR correction. No significant associations were observed for IL-33 or IL-35 after FDR adjustment, nor for HLA-DR+ or TIM-3+ subsets.

Overall, these findings suggest that plasma cytokines, particularly IL-8, are associated with γδ T cell differentiation and functional status in a compartment-specific manner, correlating with Vδ2^+^ frequencies in the placenta and with reduced activation and exhaustion marker expression in peripheral and cord blood.

Regarding memory γδ T cell phenotypes, we observed that, in peripheral blood IL-8 and IL-33 levels correlated positively with TEMRA Vδ3^+^ cells; while IL-33 levels correlated negatively with CM Vδ3^+^ frequencies (**Figure 5**). These results indicate that IL-8 and IL-33 may contribute to the differentiation of central memory γδ T cells into Terminally Differentiated Effector Memory cells in the peripheral compartment. Although several correlations reached nominal significance (p < 0.05), none remained statistically significant after correction for multiple testing using the Benjamini-Hochberg false discovery rate (FDR) correction (q ≥ 0.05).

### γδ T cell subsets are metabolically programmed in distinct ways across different blood compartments

**Figure 6:**
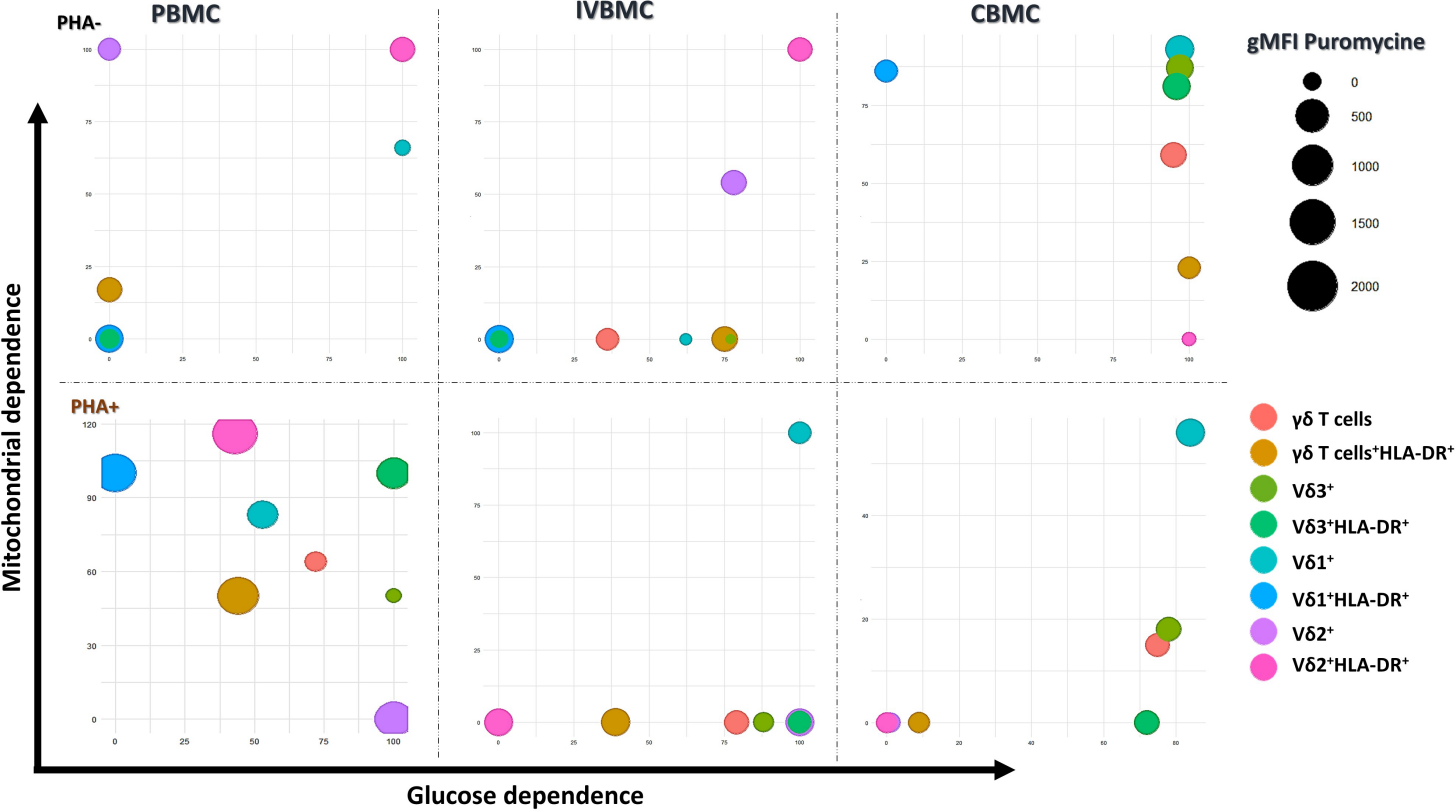
Bubble plot showing mitochondrial and glycolytic dependencies of γδ T cell phenotypes in different blood compartments. This figure shows the metabolic profiles of γδ T-cell subsets across PBMC, IVBMC, and CBMC under unstimulated (PHA−) and stimulated (PHA+) conditions. The colors represent distinct γδ T-cell subsets and bubble size reflect protein synthesis activities (puromycin gMFI). Bubble plots were generated in RStudio using the ggplot2 package. PHA-, Phytohemagglutinin unstimulated cells; PHA+, Phytohemagglutinin stimulated cells; PBMC, Peripheral blood mononuclear cell; IVBMC, Intervillous blood mononuclear cell; CBMC, Cord blood mononuclear cell.

**Figure 7:**
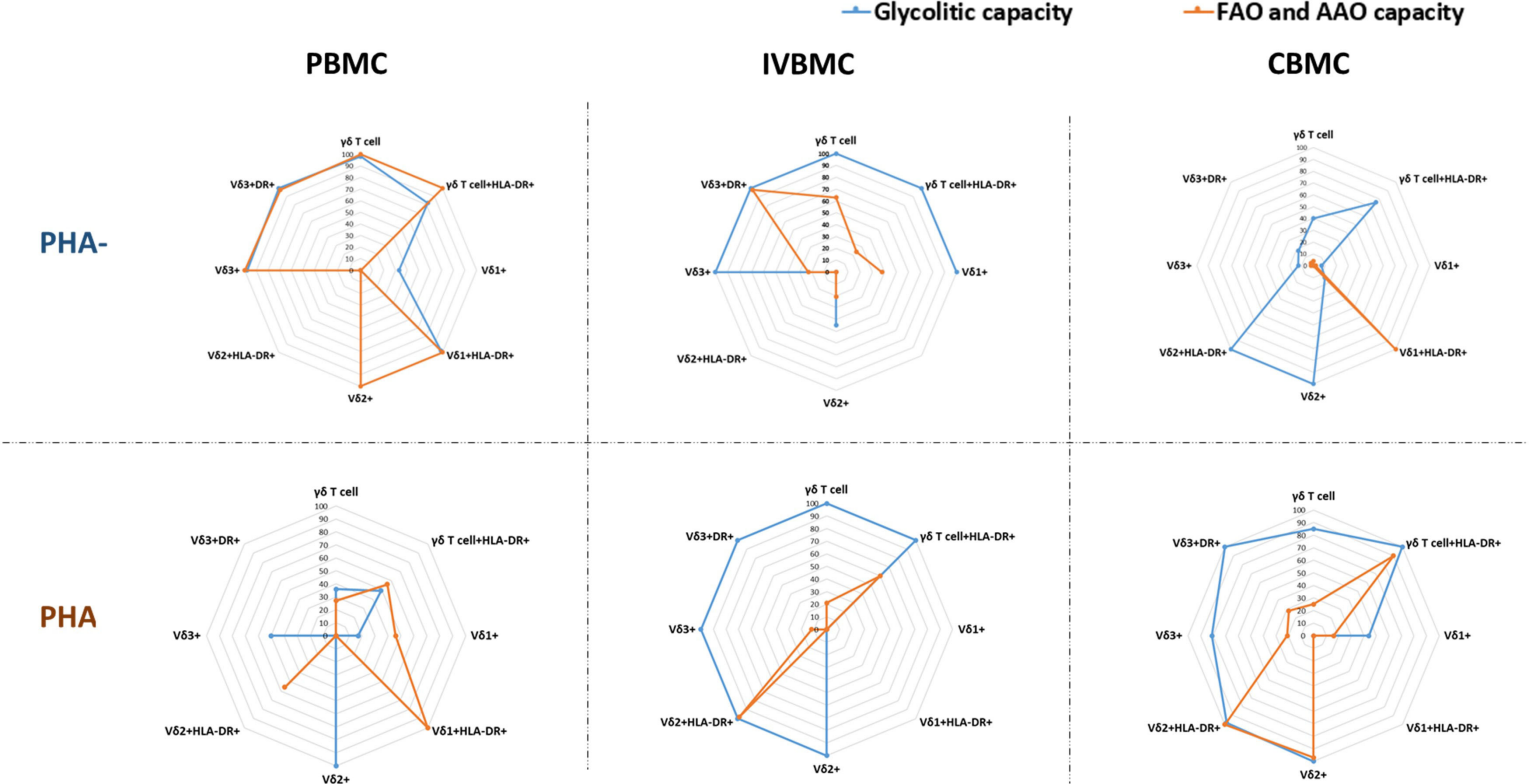
Radar plot showing glycolytic and FAO&AAO capacities of γδ T cell phenotypes in different blood compartments. This figure presents radar plots comparing the metabolic capacities of γδ T-cell subsets across PBMC, IVBMC, CBMC under unstimulated (PHA−) and stimulated (PHA+) metabolic conditions. The blue line represents glycolytic capacity, while the orange line represents fatty-acid oxidation and amino-acid oxidation (FAO/AAO) capacity. PHA-, Phytohemagglutinin unstimulated cells; PHA+, Phytohemagglutinin stimulated cells; PBMC, Peripheral blood mononuclear cell; IVBMC, Intervillous blood mononuclear cell; CBMC, Cord blood mononuclear cell.

Cellular metabolism serves as a fundamental regulator of γδ T-cell biology, and distinct γδ T-cell subsets engage specialized metabolic programs-ranging from differential utilization of glycolysis, oxidative phosphorylation, and lipid metabolism to sustain their development. As shown in **Figure 6**, Vδ2^+^HLA-DR^+^ cells in unstimulated PBMCs and IVBMCs (PHA-) utilize both glycolytic and mitochondrial pathways to support protein synthesis. In contrast, within CBMCs, Vδ2^+^HLA-DR^+^ cells depend exclusively on glycolysis, whereas Vδ1^+^HLA-DR^+^ cells rely predominantly on mitochondrial energy metabolism. In PBMCs, cell stimulation by phytohemagglutinin (PHA+) induces heterogeneity in metabolic energy requirement in different subsets of γδ T cells, with mainly mitochondrial energy metabolism activated in Vδ1^+^HLA-DR^+^ cells, both mitochondrial and glycolytic energy dependence activated in Vδ3^+^ HLA-DR^+^ cells, and a shift from mitochondrial dependence to exclusively glycolytic dependence in Vδ2^+^ cells (**Figure 6**). Conversely, in IVBMC and CBMC, the activation of both mitochondrial and glycolytic dependence was observed in Vδ1^+^ cells, while a shift from both mitochondrial and glycolytic dependence to mainly glycolytic dependence was observed for total γδ T cells, Vδ3^+^ cells, and HLA-DR^+^ Vδ3^+^ cells. Furthermore, as shown in **Figure 7**, cell stimulation with PHA differentially modulated glycolysis, fatty acid oxidation (FAO), and amino acid oxidation (AAO) in the different γδ T cells subsets in all three blood compartments (PBMC, IVBMC, CBMC). Overall, the results show that γδ T cells exhibit metabolic plasticity, adopting a glycolysis-dominant strategy in fetal compartments and a mixed metabolic profile in peripheral blood to support prolonged and diverse immune functions.

### Association of memory γδ T cell frequency with maternal and fetal outcomes

**Figure 8:**
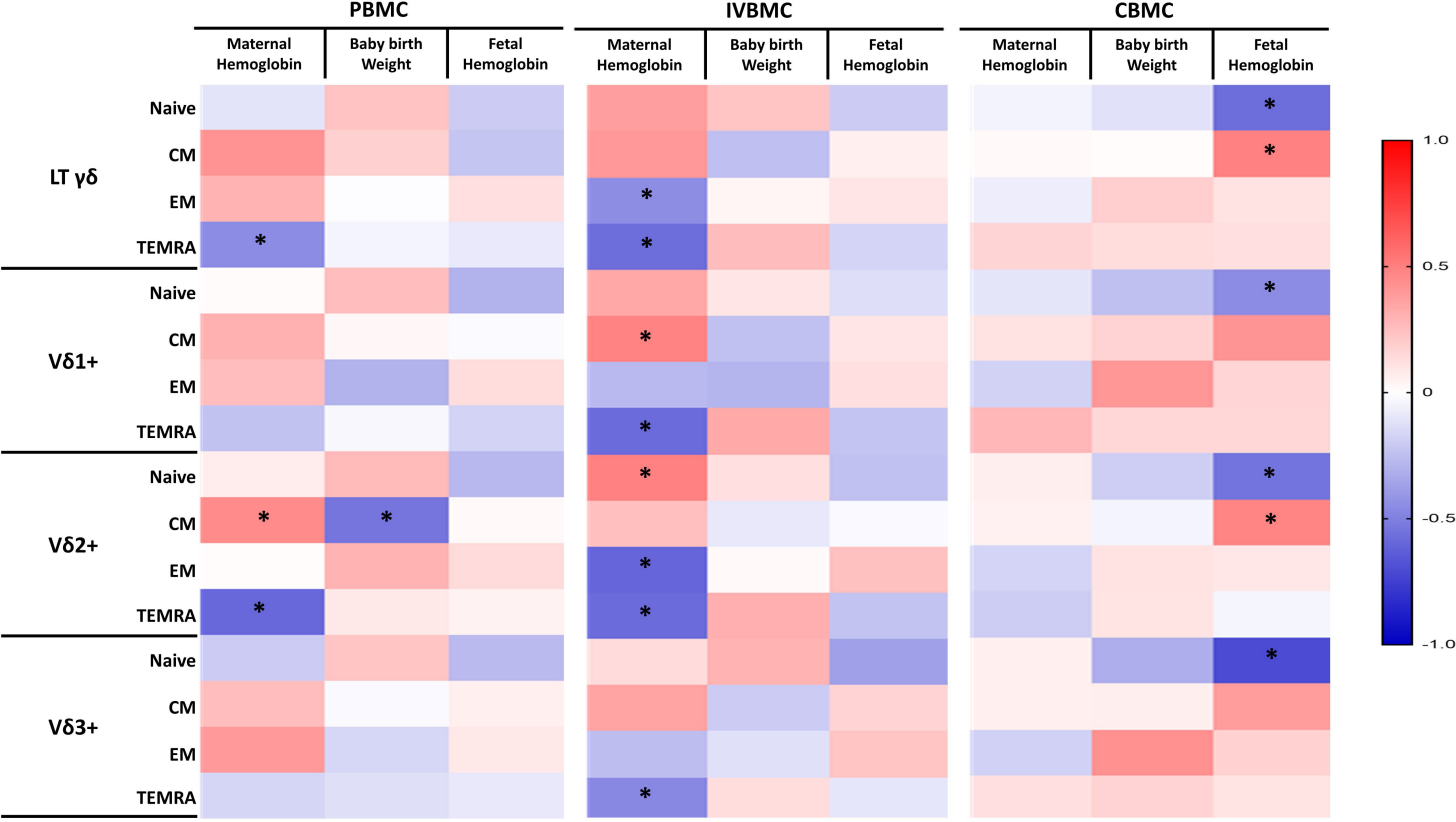
Heatmap depicting the correlation between memory γδ T cells in PBMC, IVBMC, CBMC and maternal hemoglobin level, baby birth weight, and fetal hemoglobin levels. Correlation between memory γδ T cell frequency and pregnancy outcome in different blood compartments were determined by Sperman’s rank Order correlation analysis. PBMC, Peripheral blood mononuclear cell; IVBMC, Intervillous blood mononuclear cell; CBMC, Cord blood mononuclear cell; Naïve cells, CD45RA^+^CD27^+^; CM (Central Memory) cells, CD45RA^-^CD27^+^; EM (Effector Memory) cells, CD45RA^-^CD27^-^; TEMRA (Terminally Differentiated Effector Memory) cells: CD45RA^+^CD27^-^. The asterisks indicate significance. Color intensity indicates correlations: red indicates positive correlations, white indicates no correlation, and blue indicates negative correlations. To control for false discoveries arising from multiple testing, the Benjamini-Hochberg false discovery rate (FDR) correction was applied across all performed correlation analyses. Adjusted q-values < 0.05 were considered statistically significant.

In PBMC, TEMRA γδ T cells and the Vδ2^+^ subset frequencies correlatednegatively with maternal hemoglobin levels (**Supplementary Table 1**), whereas CM Vδ2^+^ cells correlated positively with maternal hemoglobin levels but negatively with birth weight. In IVBMC, naïve Vδ2^+^ and CM Vδ1^+^ correlated positively with maternal hemoglobin levels, while EM γδ T cells and the Vδ2^+^ subset, as well as TEMRA γδ T cell and subsets (Vδ1^+^, Vδ2^+^, and Vδ3^+^), increased with decreasing maternal hemoglobin levels (**Supplementary Table 2**). In CBMC, naïve γδ T cells and subsets correlated negatively with fetal hemoglobin levels, while CM γδ T cells and Vδ2^+^ increased with increasing fetal hemoglobin levels (**Supplementary Table 3**). Following FDR correction, only TEMRA γδ T cells and their subsets (Vδ1^+^, Vδ2^+^), EM Vδ2^+^ cells in IVBMC, and naïve Vδ3^+^ cells in CBMC remained significantly negatively correlated with maternal and fetal hemoglobin levels, respectively (**Figure 8**; **Supplementary Tables 1**–**S3**). Overall, these results suggest EM and TEMRA subsets may be associated with increased risk of anemia.

## Discussion

γδ T cells play a pivotal role in the immune response to malaria, particularly in children under five years of age and in pregnant women at delivery. While emerging evidence highlights the potential of γδ T cells as targets to enhance vaccine efficacy through their regulatory and cytotoxic functions, the mechanisms underlying their differentiation remain poorly understood particularly in the context of placental malaria. Our data show that the frequencies of naïve and central memory (CM) γδ T cells, including its subset (Vδ1^+^, Vδ2^+^ and Vδ3^+^) were highest in umbilical cord blood, followed by placental intervillous space blood. In contrast, the frequencies of Effector memory (EM) and Terminally Differentiated Effector Memory (TEMRA) γδ T cells and its subsets were greater in peripheral blood followed by placental intervillous blood space. This distribution pattern suggests a compartmentalization of memory γδ T cell subsets according to their differentiation and functional state, consistent with previous findings on CD4+ T cells (27, 28). The results also suggest that maternal and cord blood compartments are programmed differently, and that neonates are characterized by a higher proliferative capacity than pregnant women during infection. The predominance of naïve and central memory γδ T cells in umbilical cord and placental intervillous blood indicates a less differentiated immune environment at the maternal fetal interface, possibly reflecting limited antigen exposure during pregnancy. In contrast, the higher frequencies of EM and TEMRA γδ T cells in peripheral blood suggest a more experienced and functionally active immune profile in the mother, consistent with repeated antigenic stimulation such as during exposure to malaria parasites.

Following *Plasmodium falciparum* placental infection, the frequencies of naïve γδ T cells as well as naïve Vδ1^+^, Vδ2^+^, Vδ3^+^ cells were significantly lower in PM+ compared to PM- women. In contrast, EM γδ T cells and its subsets were generally found at higher frequencies across all three compartments in PM+ women. These findings are similar to those obtained by Cairo et al. (29), where a predominance of the Central Memory Vγ2^+^Vδ2^+^ subset in CBMCs from PM+ women was demonstrated. In our study, CM γδ T cells and its subsets were equally predominant in cord blood from PM+ compared to PM- women. Overall, these findings indicate a shift in γδ T-cell differentiation from a naïve toward a more differentiated effector memory phenotype during *Plasmodium falciparum* placental infection, presumably resulting from ongoing antigenic stimulation and γδ T-cell activation. Although, in this study, malaria parasites could not be observed microscopically in cord blood, it is possible that phosphoantigens known to activate Vγ9Vδ2 T cells (30) may have crossed the feto-placental barrier to stimulate cord blood γδ T cells.

Chronic activation of immune cells can be regulated by multiple mechanisms, including cytokine signaling, energy metabolism and the expression of immunoregulatory markers. Our results showed that, cytokine levels (IL-8, IL-33 and IL-35) quantified ex vivo correlated negatively with the expression of the immunoregulatory markers PD-1 and TIM-3 within γδ T cells, including its subsets (Vδ1^+^, Vδ2^+^ and Vδ3^+^). Moreover, IL-8 and IL-33 levels exhibited negative correlations with Vδ1^+^ frequencies, but positive correlations with Vδ2^+^ frequencies. Although these associations did not remain statistically significant after FDR correction, the magnitude of the correlation coefficients remained high. Together, these findings suggest that immunoregulatory cytokines generated during *Plasmodium falciparum* placental infection could drive γδ T-cell activation and differentiation. The negative correlations between cytokine levels and PD-1/TIM-3 expression indicate that higher cytokine production is associated with reduced immune inhibition, reflecting a more activated immune state. Our results are consistent with those of Zhao et al., who demonstrated that macrophages-derived IL8 inhibits CD8^+^ T-cell function by downregulating TIM3 expression through IL8-CXCR2 axis in patients with advanced colorectal cancer (31). Furthermore, the negative correlation between IL-8 and IL-33 and Vδ1^+^ cells, as opposed to a positive correlation with Vδ2^+^ cells, suggests that these cytokines may differentially associate with the activation/expansion of Vδ2^+^ T cells, while limiting Vδ1^+^ responses, thereby contributing to the compartmentalized modulation of γδ T cell immunity at the maternal-fetal interface. Beyond cytokines shaping T-cell differentiation, the availability of nutrients can profoundly influence their function by redirecting metabolic pathways (23). The energy metabolism of γδ T cells is closely linked to their activation and cytokine production (32), allowing them to meet the energetic demands of their environment. We show for the first time that normal unstimulated γδ T cells exhibit blood compartment- and subset-specific metabolic profiles, with Vδ2^+^HLA-DR^+^ cells relying on glucose and mitochondrial pathways in both PBMCs and IVBMCs, whereas in CBMCs, Vδ2^+^ cells are glycolytic while Vδ1^+^ cells are mitochondrial-dependent. Upon stimulation, a majority of γδ T cell subpopulations switch between the glycolytic and mitochondrial pathways, indicating high metabolic plasticity in response to environmental and antigenic cues. These adaptations likely reflect functional differences, particularly in terms of cell activation, cytokine production, and effector response processes, between different blood compartments and γδ T cell subsets. Changes in metabolic pathways and cytokine profiles may be associated with adverse or favorable pregnancy outcomes. Indeed, consistent with our previous findings on the dichotomous functions of γδ T cells (19), we observed that Effector Memory and Terminally Differentiated Effector Memory γδ T cells and subsets (Vδ1^+^, Vδ2^+^ and Vδ3^+^) in PBMC and IVBMC were negatively associated with maternal hemoglobin levels. In contrast, naive Vδ2^+^ subset and Central Memory Vδ1^+^ subset in IVBMC associated positively with maternal hemoglobin levels. Interestingly, in CBMCs, we observed strong association between fetal hemoglobin levels and the frequencies of naïve and central memory γδ T cells and subsets. Taken together, these findings suggest that CM γδ T cell subsets may protect against maternal and fetal anemia, while EM and TEMRA subsets may favor pregnancy-associated anemia.

Although phenotypes of memory γδ T cells and its subsets in placental malaria have been characterized for the first time in this study, as well as their involvement in pregnancy outcome, several limitations should be acknowledged. First, there is lack of data on the presence of antigenic stimulants in cord blood, which could lead to a better understanding of the differentiation of memory γδ T cells in this compartment. Second, the lack of in-depth functional data on γδ T cell responses, limits full understanding of the link between cytokines produced by different memory cell types and pregnancy outcomes. Third, a surrogate gating strategy, potentially including other CD3^+^TCRγδ^+^Vδ1^−^Vδ2^−^ cell populations, was employed to define the Vδ3 cell subsets.

## Conclusion

This study provides for the first time ex vivo evidence of the interplay between γδ T-cell differentiation, cytokine responses, and metabolic reprogramming during placental infection by *P. falciparum*. The study further highlights the critical role of cellular immune response processes in shaping pregnancy outcomes. Additionally, the study implicates immunoregulatory cytokine responses and key cellular metabolic pathways in the activation and functioning of γδ T cells, which could have implications for the management of *Plasmodium falciparum* placental malaria.

## Data Availability

The original contributions presented in the study are included in the article/**Supplementary Material**. Further inquiries can be directed to the corresponding author.

## Supplementary material

Supplementary Table 1 Correlations between memory γδ T cells in PBMC and pregnancy outcomes. Correlation between memory γδ T cell frequency and pregnancy outcome in peripheral blood were determined by Sperman’s rank Order correlation analysis. PBMC: Peripheral blood mononuclear cells, Hb: Hemoglobin, Naïve cells: CD45RA^+^CD27^+^, CM (Central Memory) cells: CD45RA^-^CD27^+^, EM (Effector Memory) cells: CD45RA^-^CD27^-^, TEMRA (Terminally Differentiated Effector Memory) cells: CD45RA^+^CD27^-^. r_s_: Spearman’s rank correlation coefficient, p value: probability value. p-values were obtained from Spearman correlation tests. q-values represent p-values adjusted for multiple comparisons using the Benjamini-Hochberg false discovery rate (FDR) method. Statistical significance was defined as q < 0.05.

Supplementary Table 2 Correlations between memory γδ T cells in IVBMC and pregnancy outcomes. Correlation between memory γδ T cell frequency and pregnancy outcome in intervillous space blood were determined by Sperman’s rank Order correlation analysis. IVBMC: intervillous space blood mononuclear cells, Hb: Hemoglobin, Naïve cells: CD45RA^+^CD27^+^, CM (Central Memory) cells: CD45RA^-^CD27^+^, EM (Effector Memory) cells: CD45RA^-^CD27^-^, TEMRA (Terminally Differentiated Effector Memory) cells: CD45RA^+^CD27^-^. r_s_: Spearman’s rank correlation coefficient, p value: probability value. p-values were obtained from Spearman correlation tests. q-values represent p-values adjusted for multiple comparisons using the Benjamini-Hochberg false discovery rate (FDR) method. Statistical significance was defined as q < 0.05.

Supplementary Table 3 Correlations between memory γδ T cells in CBMC and pregnancy outcomes. Correlation between memory γδ T cell frequency and pregnancy outcome in cord blood were determined by Sperman’s rank Order correlation analysis. CBMC: Cord blood mononuclear cells, Hb: Hemoglobin, Naïve: CD45RA^+^CD27^+^, CM (Central Memory) cells: CD45RA^-^CD27^+^, EM (Effector Memory) cells: CD45RA^-^CD27^-^, TEMRA (Terminally Differentiated Effector Memory) cells: CD45RA^+^CD27^-^. r_s_: Spearman’s rank correlation coefficient, p value: probability value. p-values were obtained from Spearman correlation tests. q-values represent p-values adjusted for multiple comparisons using the Benjamini-Hochberg false discovery rate (FDR) method. Statistical significance was defined as q < 0.05.
