# Supplementary figures and images for: Ex vivo cytokine responses and metabolic reprogramming are associated with γδ T cell differentiation in Plasmodium falciparum placental malaria
Source: Front Immunol. 2026 Apr 16;17:1803384. doi: 10.3389/fimmu.2026.1803384 (PMC13128425; doi:10.3389/fimmu.2026.1803384)

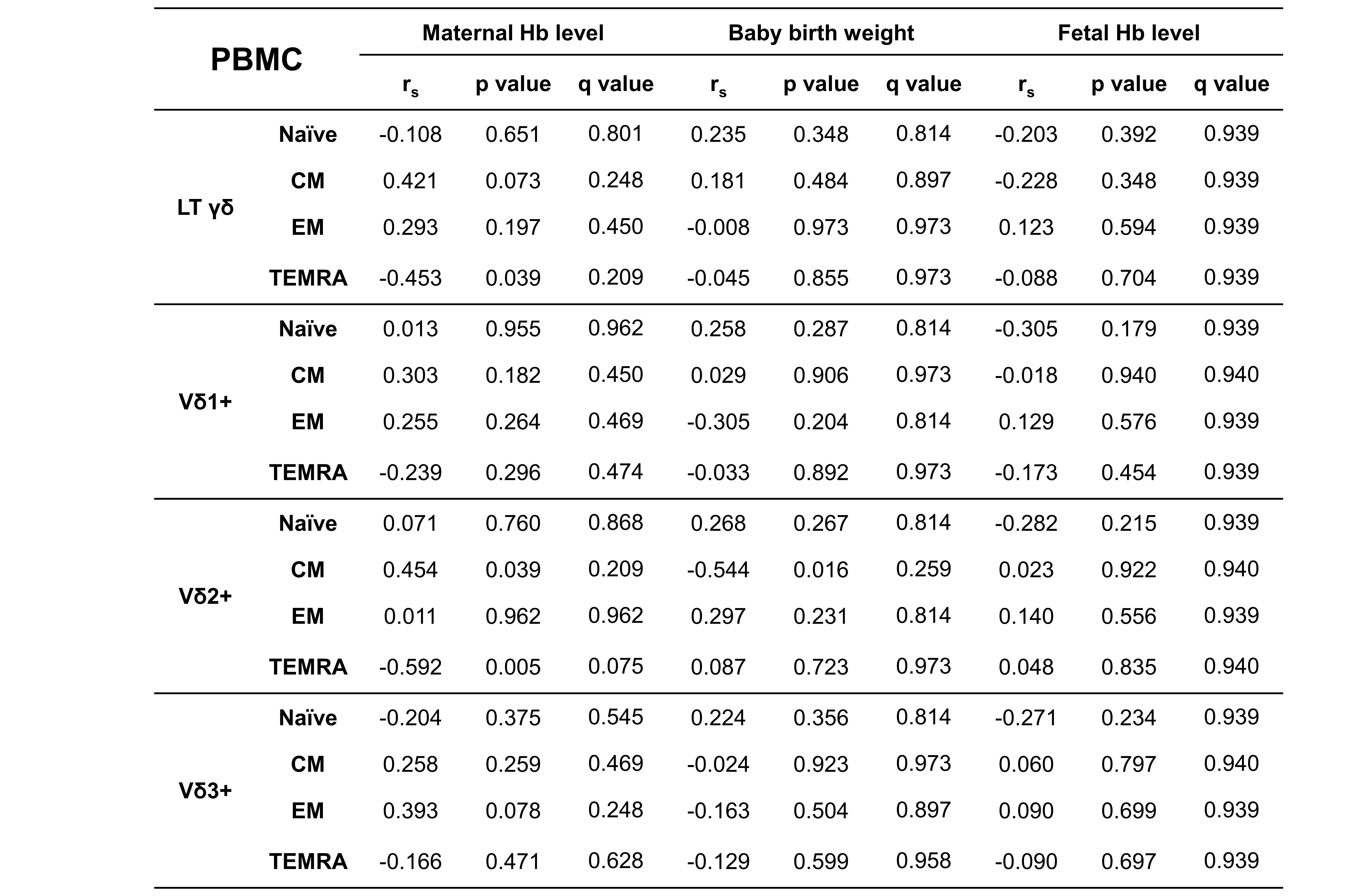

Supplement: Supplementary Table 1 — Correlations between memory γδ T cells in PBMC and pregnancy outcomes. Correlation between memory γδ T cell frequency and pregnancy outcome in peripheral blood were determined by Sperman’s rank Order correlation analysis. PBMC: Peripheral blood mononuclear cells, Hb: Hemoglobin, Naïve cells: CD45RA+CD27+, CM (Central Memory) cells: CD45RA-CD27+, EM (Effector Memory) cells: CD45RA-CD27-, TEMRA (Terminally Differentiated Effector Memory) cells: CD45RA+CD27-. rs: Spearman’s rank correlation coefficient, p value: probability value. p-values were obtained from Spearman correlation tests. q-values represent p-values adjusted for multiple comparisons using the Benjamini-Hochberg false discovery rate (FDR) method. Statistical significance was defined as q < 0.05. [file Image1.jpeg]

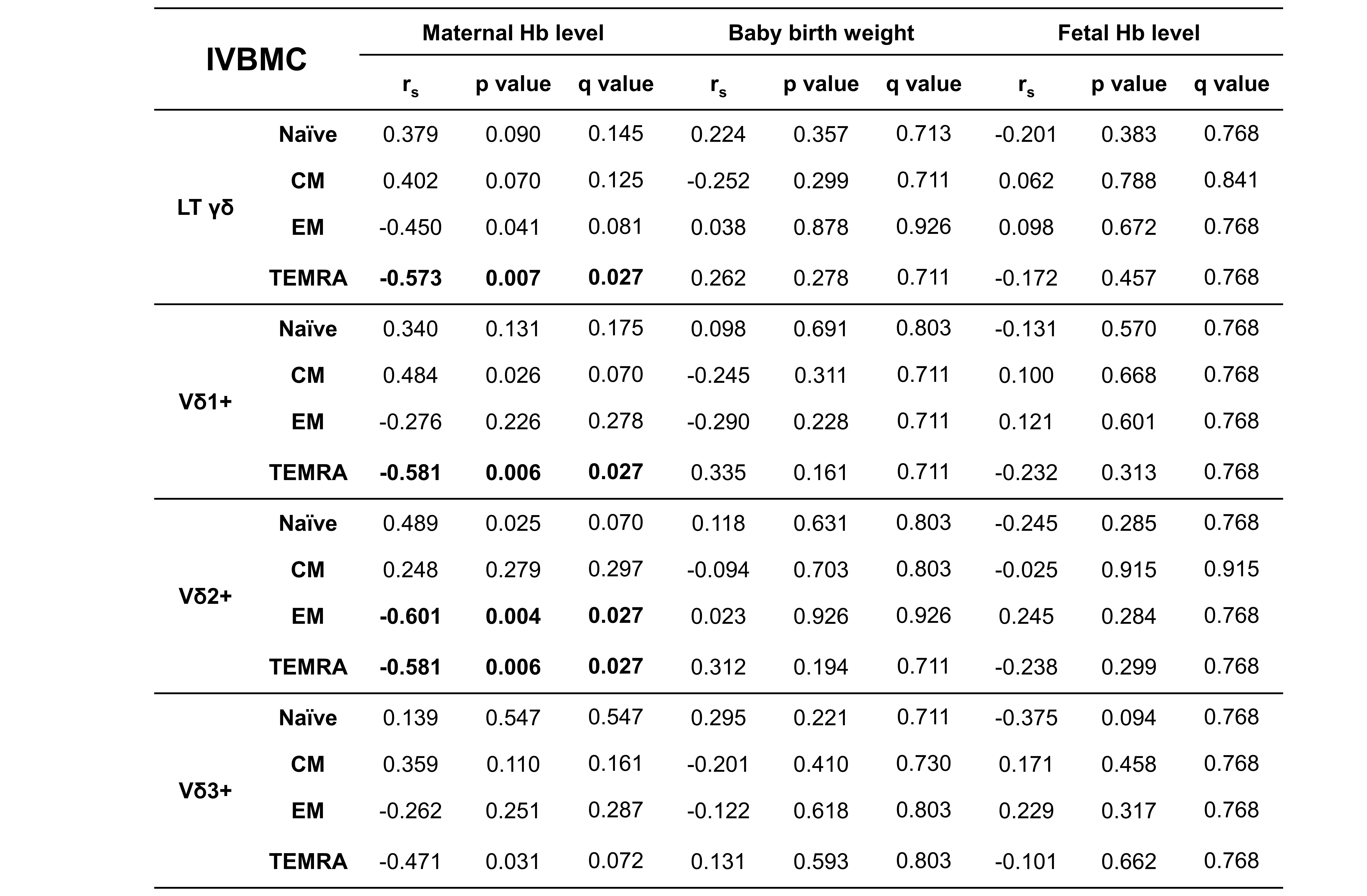

Supplement: Supplementary Table 2 — Correlations between memory γδ T cells in IVBMC and pregnancy outcomes. Correlation between memory γδ T cell frequency and pregnancy outcome in intervillous space blood were determined by Sperman’s rank Order correlation analysis. IVBMC: intervillous space blood mononuclear cells, Hb: Hemoglobin, Naïve cells: CD45RA+CD27+, CM (Central Memory) cells: CD45RA-CD27+, EM (Effector Memory) cells: CD45RA-CD27-, TEMRA (Terminally Differentiated Effector Memory) cells: CD45RA+CD27-. rs: Spearman’s rank correlation coefficient, p value: probability value. p-values were obtained from Spearman correlation tests. q-values represent p-values adjusted for multiple comparisons using the Benjamini-Hochberg false discovery rate (FDR) method. Statistical significance was defined as q < 0.05. [file Image2.jpeg]

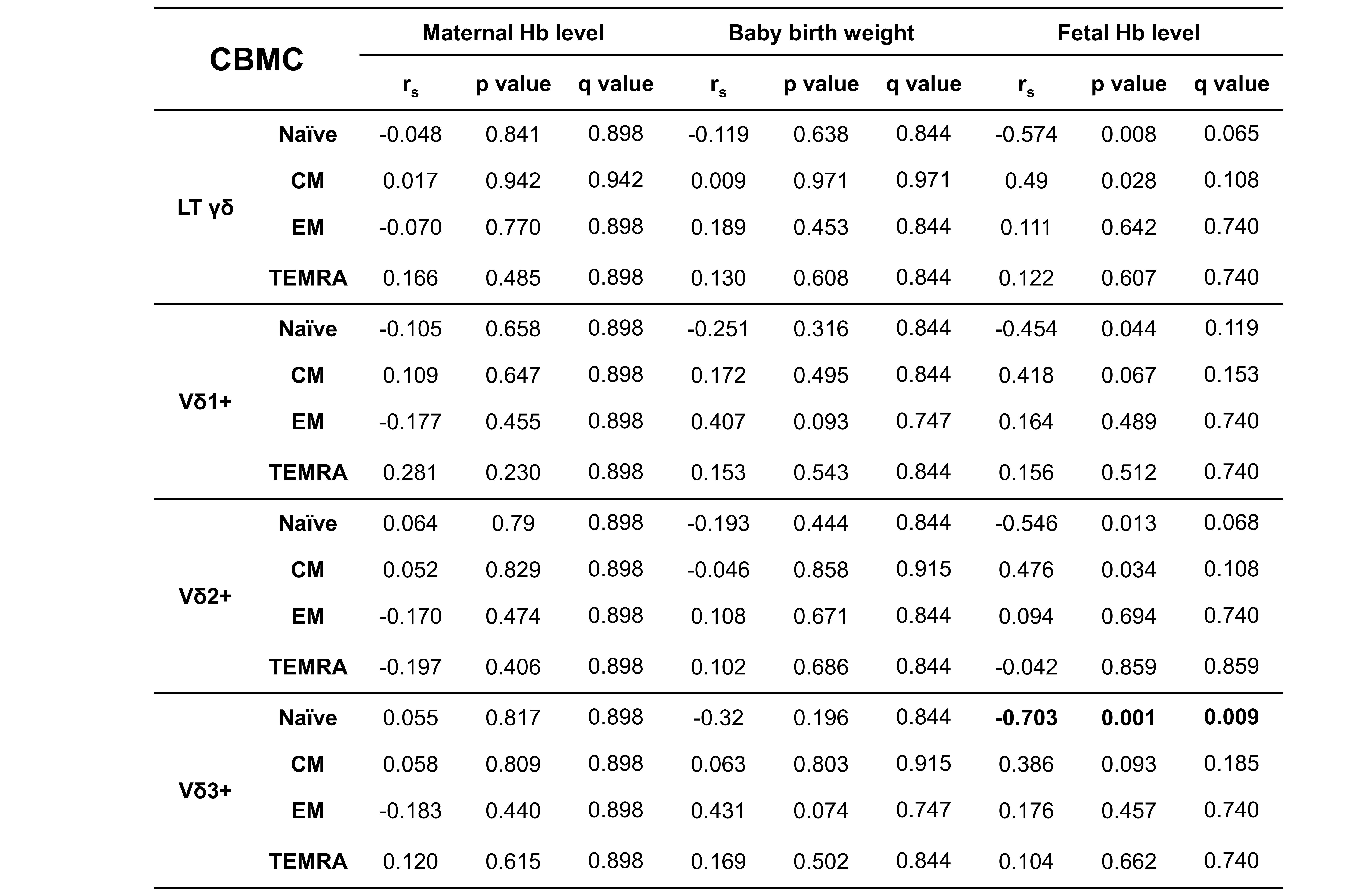

Supplement: Supplementary Table 3 — Correlations between memory γδ T cells in CBMC and pregnancy outcomes. Correlation between memory γδ T cell frequency and pregnancy outcome in cord blood were determined by Sperman’s rank Order correlation analysis. CBMC: Cord blood mononuclear cells, Hb: Hemoglobin, Naïve: CD45RA+CD27+, CM (Central Memory) cells: CD45RA-CD27+, EM (Effector Memory) cells: CD45RA-CD27-, TEMRA (Terminally Differentiated Effector Memory) cells: CD45RA+CD27-. rs: Spearman’s rank correlation coefficient, p value: probability value. p-values were obtained from Spearman correlation tests. q-values represent p-values adjusted for multiple comparisons using the Benjamini-Hochberg false discovery rate (FDR) method. Statistical significance was defined as q < 0.05. [file Image3.jpeg]
